# Guided Self-rehabilitation Contract *vs* conventional therapy in chronic peripheral facial paresis: VISAGE, a multicenter randomized controlled trial

**DOI:** 10.1186/s12883-023-03096-8

**Published:** 2023-04-10

**Authors:** Marjolaine Baude, Marina Guihard, Caroline Gault-Colas, Ludovic Bénichou, André Coste, Jean-Paul Méningaud, David Schmitz, Pierre-André Natella, Etienne Audureau, Jean-Michel Gracies

**Affiliations:** 1 BIOTN Research Unit 7377, Université Paris-Est Créteil (UPEC), 94000 Créteil, France; 2 Service de Rééducation Neurolocomotrice, AP-HP, Hôpitaux Universitaires Henri Mondor, 1 Rue Gustave Eiffel, 94000 Créteil, France; 3 Hôpital Paris Saint-Joseph, Service de Chirurgie Maxillo-Faciale Stomatologie, 75015 Paris, France; 4 Centre Hospitalier Intercommunal Créteil, Service d’ORL, Stomatologie Et Chirurgie Cervico-Faciale, 94000 Créteil, France; 5 AP-HP, Service de Chirurgie Plastique, Reconstructrice, Esthétique Et Maxillo-Faciale, Hôpitaux Universitaires Henri Mondor, 94000 Créteil, France; 6 AP-HP, Unité de Recherche Clinique, Hôpitaux Universitaires Henri Mondor, 94000 Créteil, France; 7 AP-HP, Service de Santé Publique, Hôpitaux Universitaires Henri Mondor, 94000 Créteil, France; 8 DHU A-TVB, IRMB- EA 7376 CEpiA (Clinical Epidemiology And Ageing Unit), Université Paris Est-Créteil, 94000 Créteil, France

**Keywords:** Facial paralysis, Rehabilitation, Neuronal plasticity, Efferent pathways, Guided self-rehabilitation contract, Motion capture, Three-dimensional, Creteil scale, Sunnybrook facial grading system, Patient health questionnaire

## Abstract

**Background:**

One year after persistent peripheral facial paresis (PFP), prescriptions of conventional rehabilitation are often downgraded into maintenance rehabilitation or discontinued, the patient entering what is seen as a chronic stage. This therapeutic choice is not consistent with current knowledge about behavior-induced plasticity, which is available all life long and may allow intense sensorimotor rehabilitation to remain effective. This prospective, randomized, multicenter single-blind study in subjects with chronic unilateral PFP evaluates changes in facial motor function with a Guided Self-rehabilitation Contract (GSC) *vs.* conventional therapy alone, carried out for six months.

**Methods:**

Eighty-two adult subjects with chronic unilateral PFP (> 1 year since facial nerve injury) will be included in four tertiary, maxillofacial surgery (2), otolaryngology (1) and rehabilitation (1) centers to be randomized into two rehabilitation groups. In the experimental group, the PM&R specialist will implement the GSC method, which for PFP involves intensive series of motor strengthening performed daily on three facial key muscle groups, *i.e.* Frontalis, Orbicularis oculi and Zygomatici. The GSC strategy involves: i) prescription of a daily self-rehabilitation program, ii) teaching of the techniques involved in the program, iii) encouragement and guidance of the patient over time, in particular by requesting a quantified diary of the work achieved to be returned by the patient at each visit. In the control group, participants will benefit from community-based conventional therapy only, according to their physician’s prescription. The primary outcome measure is the composite score of Sunnybrook Facial Grading System. Secondary outcome measures include clinical and biomechanical facial motor function quantifications (Créteil Scale and 3D facial motion analysis through the Cara system), quality of life (Facial Clinimetric Evaluation and Short-Form 12), aesthetic considerations (FACE-Q scale) and mood representations (Hospital Anxiety and Depression scale). Participants will be evaluated every three months by a blinded investigator, in addition to four phone calls (D30/D60/D120/D150) to monitor compliance and tolerance to treatment.

**Discussion:**

This study will increase the level of knowledge on the effects of intense facial motor streng- Facial paralysisthening prescribed through a GSC in patients with chronic peripheral facial paresis.

**Trial registration:**

ClinicalTrials.gov, NCT04074018. Registered 29 August 2019.

**Protocol version:**

Version N°4.0—04/02/2021.

**Supplementary Information:**

The online version contains supplementary material available at 10.1186/s12883-023-03096-8.

## Background

Peripheral facial paresis (PFP) is a common disorder, which often affects adults between 40 and 50, with no gender predominance. PFP results from an injury to the facial nerve, from its pontine nucleus to its neuromuscular junctions; its severity depends on lesion location, cause and degree of injury. Seventy percent of cases are idiopathic, called Bell’s palsy, with a lifetime incidence of 1 in 60 people [1], representing an incidence of 15 to 50 new cases for 100 000 persons per year [1, 2]. In 70–90% of cases, recovery to baseline occurs within 6–9 months [3–6]. The other 10–30% enter a chronic stage with one of two clinical forms: a non-overactive form with flaccidity of the paretic hemiface, described as a global loss of tone, and an overactive form mixing paresis and facial involuntary muscle overactivity in the affected hemiface [7]. Muscle overactivity may occur at rest through involuntary tonic contractions of some facial muscles, called facial dystonia, or through small brief contractions known as facial spasms. Muscle overactivity may also occur during movements, manifesting as facial cocontractions known as synkinesis. These persistent motor signs strongly impede quality of life, aesthetically and psychologically [8, 9].

Regarding clinical evaluation of PFP, heterogeneity in assessments makes comparisons between various interventions difficult [10]. The classic House-Brackmann Grading Scale is still used in clinical practice but no longer recommended due to inadequate psychometric characteristics, regarding its validity in particular [10, 11]. In 2015, the *Sunnybrook Facial Grading System* (SFGS) was suggested as a new standard in reporting outcomes of facial nerve injuries [10, 12]. Additional criteria for grading were later added to improve its reliability [13]. Nevertheless, evaluations through SFGS have four important limitations: i) the scale remains an ordinal grading system with few grades, lacking accuracy and thus jeopardizing responsiveness; ii) it investigates only five facial muscle groups, among over twenty muscle groups on each hemiface, omitting platysma for example; iii) it does not assess spontaneous and/or conversational facial movements even though this is the main function of the facial nerve, i.e. to reflect our emotions; iv) it is not systematically video-recorded, precluding retrospective checking and visual follow-ups. Recently, a Delphi study on consensus research priorities for facial palsy insisted on standardization of clinical assessments as a high priority for both patients and healthcare professionals [14].

In terms of rehabilitation, evidence from randomized controlled trials (RCT) is still insufficient, partly because of this lack of precise and objective quantification tools for facial motor function. Most publications on facial rehabilitation pertain to acute and subacute stages of PFP and involve prescriptions for physical therapy and/or speech therapy [15]. The rehabilitation techniques used are varied and heterogeneous, and consensus is lacking. A longstanding, classic principle has been to avoid muscle strengthening exercises and facial muscle electrical stimulation, especially in acute stages, for fear of generating muscle overactivity [15]. Yet, RCTs involving active motion exercises in acute stages have shown improved recovery, particularly in severe cases, with no increase—or even a decrease—in synkinesis [16, 17]. As for electrical neurostimulation of the facial nerve, despite also being controversial in acute stages [18], both classic and recent evidence from controlled literature favors either no effect or a positive effect with respect to synkinesis [19–21].

To our knowledge, there is no RCT analyzing motor strengthening in chronic stages (> 1 year since injury), when motor impairments are often considered permanent [20]. In other fields of neurorehabilitation, a considerable body of evidence shows that high intensity of rehabilitation (the opposite of “maintenance therapy”) is associated with improvement of motor function in chronic stages, via enhanced brain plasticity [22–25], specifically *behavior-induced plasticity*. The reality and potentially powerful effects of behavior-induced neural plasticity have been first demonstrated in the early twentieth century by Pavlov—under the misleading name of *conditional reflexes*—and its synaptic mechanisms have been elucidated by Kandel [26]. Behavior-induced plasticity involves changes in synaptic sensitivity, in neuronal connections or in cell numbers resulting from changes in environmental conditions, including behavioral changes, such as engagement into an intensive facial rehabilitation program. Such program may have the capacity to place cortical, sub-cortical, brainstem and peripheral nervous system regions under sufficient constraint, so as to strengthen and facilitate the command to facial muscles [25, 27]. Behavior-induced plasticity may take over after the end of the period of lesion-induced plasticity, which typically runs for few months after the lesion [28]. One way to achieve sufficient amounts of physical treatment might be to adequately *motivate and guide* the patient to practice self-rehabilitation. It has been confirmed that programs of exercises given by the therapist to be performed at home are appreciated by patients not only for the structure they give to everyday life, but also as they represent in themselves a source of motivation and hope, particularly when these programs are associated with ongoing professional support [29, 30].

The government-funded VISAGE project is a French multicentric RCT in which 82 patients with chronic stable unilateral PFP of any cause will be randomized into two six-month rehabilitation programs – motor strengthening through Guided Self-rehabilitation Contract *vs.* conventional therapy – to compare their effects on facial motor recovery. Once past the stages of lesion-induced nervous system plasticity, we hypothesize that behavior-induced plasticity will be stimulated through intensive motor strengthening rehabilitation work in chronic PFP using a Guided Self-rehabilitation Contract.

### Objectives

The primary objective of this study is to evaluate changes in subjective facial motor function after six months of Guided Self-rehabilitation Contract compared to conventional therapy alone, in chronic stages following facial nerve injury. Secondary objectives include the evaluation of differences in changes between the two treatment groups in: i) specific PFP-related quality of life; ii) overall quality of life; iii) aesthetic considerations; iv) mood; v) objective 3D facial motor function.

## Methods/design

### Ethical approval and trial registration

The VISAGE study has been designed in accordance with the Helsinki Declaration. The study protocol, patient information letter, and informed consent form have been approved by the Institutional Review Board ‘Est-III’ (Nancy, France) on October 3^rd^ 2018. The VISAGE study is registered in the ClinicalTrials.gov database (NCT04074018).

### Research design

VISAGE is a single-blind, prospective, controlled, randomized multicenter study in 82 participants with chronic unilateral peripheral facial paresis (> 1 year since facial nerve injury). The trial will be run in four tertiary centers, including maxillofacial surgery (2), otolaryngology (1) and neurorehabilitation (1) units. For all participants, the study will begin at inclusion (Day-30). At that time point, the investigator will implement computerized randomization to assign a group treatment to the patient, between Guided Self-rehabilitation Contract (GSC) and conventional therapy (CONV) alone, for six months. A delay of one month between inclusion (D-30) and the first assessment (D1) is anticipated to account for the time to find a therapist and to ensure timely rehabilitation onset (D1) for the participants in the CONV group. In the GSC group, the participants will be free to continue potential preexistent conventional therapy if there was any. The total duration of subject participation in the study is seven months. Study data will be analyzed through intention-to-treat and per-protocol analyses.

### Interventions

#### Conventional therapy

In the Conventional Physical Therapy group (CONV), the study PM&R specialist prescribes physiotherapy and/or speech therapy sessions at inclusion (D-30), for a six-month period starting at D1. For this purpose, a list of physical and speech therapists specialized in PFP rehabilitation and working in the Greater Paris area is provided to each subject. Each therapist of that list has personally agreed to potentially take care of patients of the VISAGE study in due course. Such community-based therapy sessions are universally and indefinitely covered by public health insurance in France. Whichever the study group, subjects already undergoing community-based rehabilitation are free to pursue that rehabilitation during the six months of the study. Indeed, the discontinuation of previous rehabilitation might lead to nocebo effects, which is well known in rehabilitation RCT studies [31, 32]. The treatment of the control group is intended to reflect the usual management of patients with chronic peripheral facial paresis. The physiotherapists caring for the patients in the control group are all trained in the rehabilitation of facial paresis and accustomed to treating this disorder. As mentioned above, there is no standardized rehabilitation protocol and the techniques are diverse and heterogeneous among therapists. Each practitioner in the control group therefore chooses the frequency of the rehabilitation sessions, the techniques used and the duration of each rehabilitation session. Such information will be noted however, by the study examiners to quantify and qualify the control group rehabilitation.

#### Guided self-rehabilitation contract

**Fig. 1:**
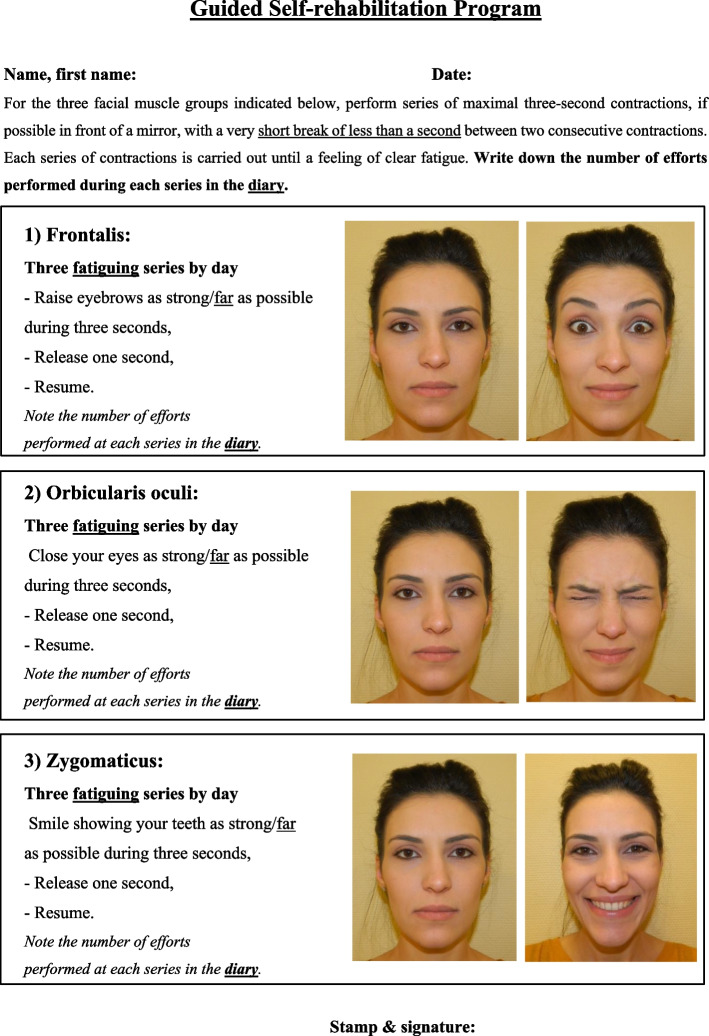
Prescription of the Guided Self-rehabilitation Contract

**Fig. 2:**
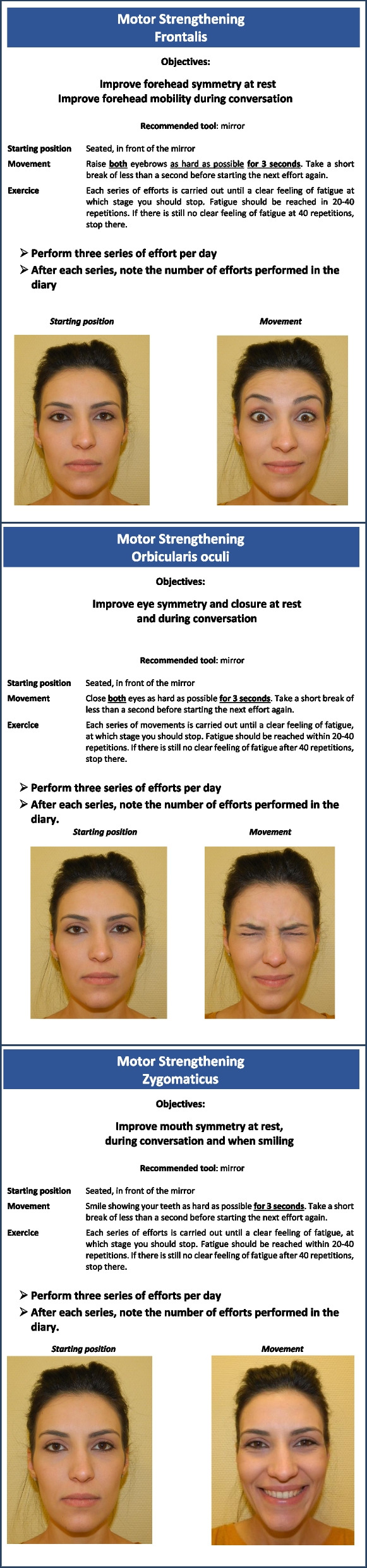
Self-rehabilitation techniques included in the Guided Self-Rehabilitation Contract

**Fig. 3:**
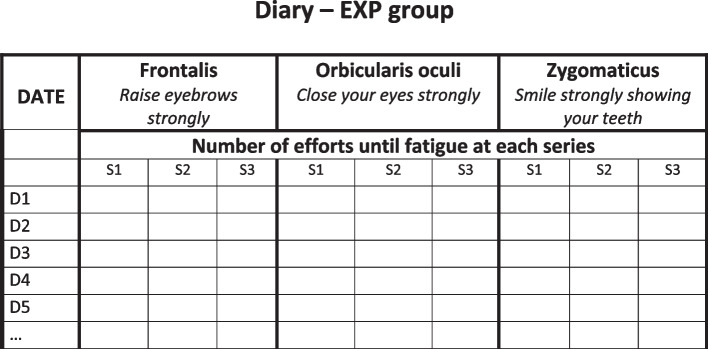
Template of diary for the Guided Self-rehabilitation Contract

In the GSC group, patients will be free to follow any conventional community-based therapy sessions, as in the CONV group. In addition, in the evaluation center, the study PM&R physician will provide three one-hour rehabilitation clinic visits at D1, D90 and D180, with the following objectives:

- *Explain the principles of the Guided Self-Rehabilitation Contract* to the patient. The GSC is a diary-based program centered on a moral contract in which each party, study participant and PM&R physician or GSC therapist, commits to the other on the following actions. The PM&R physician or GSC therapist commits to:
- *Prescribe* and *teach* a daily program of facial muscle strengthening exercises customized to the participant (see Figs. 1 and 2), and adjust the techniques according to clinical progress. Here, the study PM&R physician will provide a manual to the subject, which contains the prescribed program, with illustrations of the training exercises. Three facial muscle groups are targeted, selected because of the low risk of apraxia for the command to these muscles in the general population [33] and their localization (one for each third of the face): Frontalis, Orbicularis oculi and Zygomaticus. For each muscle group, the training program consists of three daily series of bilateral unassisted repeated efforts or movements of maximum amplitude until *fatigue* (e.g. 20 to 40 facial contractions per series, depending on fatigability). We have thus chosen to strengthen these three facial muscle groups that are crucial for facial motor function: raising eyebrows (expressiveness), closing eyes (eye protection) and smiling (communication, emotional expression, interaction with others). In practice, these muscles are easy to reach using voluntary command and therefore to rehabilitate. Each of them is located on one of the three vertical thirds of the face: during these efforts, several facial nerve branches covering the entire face are therefore stimulated from the top to the bottom of the face. Each branch of the facial nerve innervating several facial muscles, we postulate—and see in routine practice—that participants will also improve on neighboring muscles not directly targeted by the strengthening.
- *Request a diary* from the patient at each visit, in which the daily number of efforts or movements performed in each series in the interval between two visits with the PM&R physician should be recorded (Fig. 3). The PM&R physician stresses to the subject that such *self-monitoring* through the quantified written diary actually *belongs* to the therapy, in other words that the same physical exercises without maintaining the diary are likely to not carry the same efficacy [34–43]. Indeed, written feedback from patient to therapist/physician improves compliance to the self-rehabilitation program and thus the efficacy of this program [34–39]. Most importantly, the diary provides the patient with positive reinforcement, with potentially enhanced motor circuit excitability and even antidepressant effects [40–42, 44]. Regardless of self-monitoring, quantitative feedback on performance provided to the patient has been shown to improve rehabilitation effectiveness during the subacute phase of stroke [43].
- *Verify* patient compliance to the prescribed program by ensuring that the self-rehabilitation diary is well kept. The study physician will count the number of filled out days in the diary and divide it by the number of elapsed days since the last visit. In return, the subject commits to performing the prescribed daily self-rehabilitation program, including in particular the production of the written diary of exercises and to return it at each visit.

### Outcome measures

To evaluate motor function beyond one year after facial nerve injury, the primary outcome measure will be the composite score of the Sunnybrook Facial Grading System. This scale, created by Ross et al. in 1996, assesses resting symmetry compared to the normal side for each third of the face through rating of resting symmetry, of symmetry of voluntary movements on a five-grade scale during five facial movements, and associated synkinesis. A composite score is then calculated, ranging from 0 (total paralysis) to 100 (normal) [12]. The French version of the SFGS was recently validated [45]. As stated above, the choice not to use House-Brackmann evaluation scales [11, 46] is in accordance with the fact that these scales are no longer recommended in research [10, 47, 48].

Secondary outcome measures will include:

1. Specific PFP-related quality of life evaluated through the Facial Clinimetric Evaluation (FaCE), a self-questionnaire validated in French [49]. The FaCE studies specific quality of life for PFP patients through 15 items evaluating six domains [50].
2. Overall perceived quality of life evaluated through the Short Form version 12 (SF 12): this is a self-questionnaire comprising 12 items evaluating quality of life. The scoring system is ordinal. Two scores are calculated, a Physical Composite Score (PCS on 26) and a Mental Composite Score (MCS on 30) [51]; the SF 12 was validated in French in 1998 [52].
3. Aesthetic self-assessment collected using the FACE-Q scale; this is a ten-item self-questionnaire questioning the patient on facial aesthetics [53, 54]. The scoring system is ordinal from 1 (« very unsatisfied») to 4 (« very satisfied»); it has been validated in 2022 [55].
4. Anxiety and depressive disorders using the Hospital Anxiety and Depression Scale (HADS): [56]; this self-questionnaire includes 14 items rated from de 0 to 3. Seven items are related to anxiety and seven to depressive features, leading to 2 final scores (/21). Scores of 7 or less indicate a lack of symptoms, 8 to 10 suggest questionable symptomatology and 11 and more attest to definite symptomatology. The scale has been validated in French in various patient populations [57].
5. Evaluation of compliance to and amount of self-rehabilitation in the GSC group, through the filling ratio of the diary over six months.
6. Evaluation of the amount and type of conventional therapy in both groups, through collected data on the mean frequency and techniques used over six months.
7. Subjective facial motor function quantification during the Créteil Scale. The Créteil Scale (CS) has been developed by Gracies and Baude in 2012 [58] to assess 12 groups of facial muscles: frontalis, corrugator, procerus, orbicularis oculi, canine, zygomaticus, risorius, buccinator, orbicularis oris, depressor anguli oris, mentalis, and platysma. For each of these 12 muscle groups, the scale quantifies five fundamental components of facial nerve injury, at rest, during maximal effort and during spontaneous conversation: 1) *Motor strength* of the 12 facial muscle groups rated through a 9-grade scoring system from 0 (no contraction) to 3 (normal contraction); 2) Localization and intensity of involuntary *synkinesis* generated during maximal voluntary effort directed to each of the facial muscle groups; 3) Localization and frequency of involuntary *spasms* during a 30-s resting period *and* during a 30-s spontaneous conversation period, rated through an ordinal 7-grade scoring system; 4) A *paresis-related symmetry score*, quantifying symmetry at rest and during conversation searching for lacks of contraction of each muscle group; 5) A *dystonia-related symmetry score*, quantifying symmetry at rest and during conversation searching for overactivity in each muscle group. This study will explore the validity, intra- and inter-rater reliability of the Créteil Scale, as well as its responsiveness to rehabilitation in a chronic stage.
8. Objective 3D facial motor function quantification through 3D wearable (helmet) facial motion analysis through the Cara™ system. Asymmetry ratios and Procuste analysis will be performed at rest (during a 30-s period), during twelve maximal movement efforts and during spontaneous smile [59]. Measurement of the amount of synkinesis during voluntary movement, of spasms and dystonia at rest will also be performed using Procuste distances: synkinesis (quality, frequency and severity) will be assessed during each maximal voluntary movement (12 movements in total) and during one spontaneous smile; spasms will be assessed during a 30-s period of rest, as well as dystonia.

### Setting and recruitment

This multicenter trial involves four French centers: the Neurorehabilitation and the Maxillo-facial & Plastic Surgery departments at *Henri Mondor* University Hospitals, Créteil, the Maxillo-facial Surgery & Stomatology department at *Saint-Joseph* Hospital, Paris and the ENT, Stomatology & Head—Neck Surgery department at *Centre Hospitalier Intercommunal*, Creteil. Each of these centers admits > 20 cases/year of patients with unilateral peripheral facial paresis and should recruit over 20 participants in the study. A three-year period is planned for the study, each center starting the study once the total expected number of participants to be enrolled are lined up. Therefore, the objective is to run the protocol simultaneously for all participants of a given center.

### Procedures

This is a single-blind controlled trial, with randomization into two parallel groups, inclusion and randomization being computerized online (Cleanweb Telemedecine Technologies, France) in each investigator center. Clinical assessments, video recordings and 3D analyses of included subjects will be performed in a single investigating center: the Neurorehabilitation department at Henri Mondor University Hospitals, Créteil, France (evaluation center).

Screening visits will take place during regular clinic visits in each center. Participants meeting the selection criteria (see Table 1) will be invited to participate in the protocol. Written descriptive documentation will be provided and potential study subjects will be given at least two weeks to decide about participation. Informed consent will be signed and collected at D-30 of the study.

**Table 1:** Inclusion and exclusion criteria

SELECTION CRITERIA
**Inclusion criteria:**
- Chronic and stable unilateral peripheral facial paresis, *i.e., more than* one year since the onset of the symptom, whatever its cause
- Age ≥ 18
- Outpatient
- Sufficient motivation level to participate in a 6-month facial rehabilitation program, based on the opinion of the investigator
- Signed consent
- Medically insured
**Exclusion criteria:**
- Evolving tumor causing the peripheral facial paresis
- Facial botulinum toxin injection less than six months before enrollment in the study, or planned during the study period
- Facial surgery less than two years before enrollment into the study or planned during the study period
- V-VII or XII-VII anastomosis or muscle transfer prior to enrollment into the study or planned during the study
- Nonsurgical aesthetic treatments – hyaluronic acid / lipofilling / laser / tensor thread – less than two years before enrollment in the study or planned during the study period
- Recurrent facial paresis
- Participation in another interventional research protocol on the face (aesthetic, rehabilitative, or surgical)
- Intercurrent disorder that could hinder the ability to participate in the rehabilitation program
- Preexisting cognitive, mental or psychological disorder preventing participation in the rehabilitation program or in the follow-up assessments, based on the opinion of the investigator
- Patient under legal protection

Randomization will be performed on inclusion day (D-30) by the chief investigator of each center, between the Conventional therapy group (CONV) and the Guided Self-rehabilitation Contract (GSC) group (see Randomization procedure below). Each participant will thus be randomly assigned to one of the two arms and will receive a rehabilitation program depending on the randomized arm, to be carried out over six months. In order to minimize any nocebo effect for the conventional treatment group, the study coordinator will not disclose details of the Guided Self-rehabilitation Contract to the physicians and physiotherapists who treat participants in the CONV group, nor to the CONV participants themselves.

Facial rehabilitation will begin the day after the first evaluation (D1).

In practice, two PM&R neurorehabilitation study physicians, a blinded assessor and an unblinded rehabilitation prescriber and coach, will separately meet each participant at D1, D90 and D180, for an assessment visit and a rehabilitation visit: 1) during the assessment visit, the blinded evaluator will run the clinical scales, self-questionnaires, and the 3D facial analysis (except for D90); 2) during the rehabilitation clinic visit, the unblinded PM&R physician will organize the rehabilitation. After receiving the result of the randomization at D-30, he/she will: 1) inform the participant on the arm that has been randomized; 2) teach the GSC, for those participants randomized in the GSC group; 3) find a community-based therapist through a dedicated listing, for those participants randomized in the CONV group; 4) give a rehabilitation booklet to the participant, according to the arm being randomized; 5) schedule the first visit (D1). Table 2 displays the study schedule.

**Table 2:** Study schedule: enrolment, interventions and assessments

	**D-30**	**D1**	D30 ± 3 days	D60** ± **3 days	**D90 ± 2 weeks**	D120 ± 3 days	D150** ± **3 days	**D180 ± 2 weeks**
Signature of consent form	x							
Inclusion criteria checking	x							
Randomization	x							
Clinical examination	x	x			x			x
Teaching and then supervision of the guided self-rehabilitation contract (GSC group)		x			x			
Sunnybrook Facial Grading Scale	x	x			x			x
Créteil Scale		x			x			x
FaCE		x			x			x
FACE-Q		x			x			x
HADS		x			x			x
SF-12		x						x
3D facial motion analysis		x						x
Collection of adverse events					x			x
Phone calls			x	x		x	x	
Review of the diary (GSC group)					x			x

### Randomization procedure

The randomization list will be computer-generated by a statistician from the Clinical Research Unit of *Henri Mondor University Hospitals* (Créteil, France) independent of the study, and uploaded in an online case report form (electronic CRF, eCRF—Cleanweb Telemedecine Technologies, France). The chief investigator of each center will connect to the eCRF via a web browser, fill in the participant characteristics and assign participants to their study group online. A unique allocation study number will be assigned to each study participant in sequential order (R00X format). Randomization will be perform at D-30, and each participant will be assigned to one of the two treatment groups: CONV group (Conventional Therapy) or GSC group (Guided Self-rehabilitation Contract). A confirmation email will be sent to the unblinded investigator of the coordinating center. As assessment visits will be conducted in only one of the four inclusion centers, randomization will not be stratified by center. Randomization will be stratified according to the initial voluntary symmetry score of the Sunnybrook Facial Grading Scale (ranging from 5 to 25), performed at D-30. To ensure comparability of patients with respect to their level of motor function (severity) at inclusion two ranges of score will be selected: [5-15] and [16-25] for stratified randomization. Blinding will be preserved until the database is frozen.

### Study population

Eligible patients must be diagnosed with stable, chronic,—*i.e.* that occurred at least one year before inclusion—unilateral peripheral facial paresis, whatever its cause. To participate in the study, subjects must meet all other inclusion criteria and none of exclusion criteria listed in Table 1. Patients may be enrolled in this study as early as one year after onset for the following reasons: there is no consensus on the transition time to the chronic form of facial paralysis, mentioned from 6 months to two years depending on reports [60, 61]; it is often considered that most of the neurological recovery occurs within the first year after the onset even though there is no scientific evidence to date; in the same way it is considered that the further away from the onset of the facial paresis, the less the amount of conventional rehabilitation; patients are generally offered to start botulinum toxin injections from one year post onset of paresis and once these are started, it is rare for the patient to be willing to stop them, even temporarily. In Beurskens et al., 2006, it was also considered that patients 9–12 months from onset were in the chronic stage [62].

### Data management

All information required by the protocol will be entered in the eCRF. Data will be collected as they are obtained. Any missing data will be coded. Every site will have access to the eCRF via a web-based data collection system. Investigators will be given a document offering guidance on using this tool. Each investigator will be responsible for the accuracy, quality and relevance of all the data entered. An audit trail of all changes will be saved. The computer file used for this research is implemented in accordance with French (amended “Informatique et Libertés” law governing data protection) and European (General Data Protection Regulation – GDPR) regulations. The sponsor has obtained the authorization of the CNIL (“*Commission Nationale de l’Informatique et des Libertés*”, French Data Protection Agency) before implementing any of the data processing required to conduct the research. If a subject stops participating or withdraws consent, the data collected up to the date of cessation will be analyzed. If a subject is lost to follow-up, the investigator should make every effort to reconnect with the patient. Subjects who have prematurely stopped their participation in the research will not be replaced.

### Data monitoring

A Clinical Research Associate (CRA) appointed by the sponsor will be responsible for the proper processing of the study, for collecting, documenting, recording and reporting all handwritten data, in accordance with the Standard Operating Procedures applied within the ‘DRCI’ (Clinical Research and Innovation Department at *Assistance Publique-Hôpitaux de Paris*) and in accordance with Good Clinical Practices as well as with the statutory and regulatory requirements. Computerized quality control will be put in place for missing data detection and consistency of data timing. In case of incorrect filling out of the e-CRF, the investigator will be asked to correct.

### Harms

In this study involving human participants (as defined in category 2 of art. L1121-1 of the French Public Health Code), adverse events (serious or not) will not be notified to the sponsor. Notification will be implemented within the framework of the vigilance set up in usual care procedures. No data monitoring committee is needed.

### Audit

In accordance with Good Clinical Practice, the sponsor is responsible for obtaining the agreement of all parties involved in the research to ensure direct access to all research sites, source data, source documents and reports for quality control and audit purposes by the sponsor. Investigators will make the documents and individual data available to persons in charge of monitoring and quality control, in the event of an audit, in accordance with the legislative and regulatory provisions in force.

### Statistical methods

#### Sample size

The required number of subjects has been determined to be 82, from preliminary data in literature: to our knowledge the sole RCT about rehabilitation in subacute/chronic stages of PFP (participants enrolled from 9 months into the onset of paresis) using SFGS included 50 participants, among whom only 24 benefited from rehabilitation (waiting list without any rehabilitation in the other group) [62].

In contrast with that trial, participants of the control group of the present study will benefit from an intervention (physiotherapy and/or speech therapy), which will be associated with the potential to improve clinical status. We thus hypothesize that there will be a minor improvement of + 10 points in the Sunnybrook composite score in that group. Based on that hypothesis and to demonstrate an improvement of + 20 points (with a standard deviation up to 13) for the experimental group, considering a 5% alpha risk (bilateral test) and a statistical power of 90%, we will include 41 patients per group for 36 analyzed (assuming a 10% loss of follow-up).

Regarding the secondary objectives, it should be noted that this sample size will be comparable to other reliability and validity studies for the FaCE scale (*n* = 67 to 122 patients; [47, 48, 59–61] and larger than the initial Sunnybrook validation study (*n* = 19; 12). Regarding reliability parameters such as the intraclass correlation coefficients (ICC) and for ICC values expected to be > 70%, the suggested sample size will allow estimating ICCs with an accuracy of ± 15% for two measures and ± 10% for three measures and more [63]. Strategies for achieving adequate participant accrual to reach the target sample size will involve communication on the study with ENT specialists, speech therapists and physiotherapists in the greater Paris area.

#### Statistical analysis

Descriptive statistical analysis will be carried out to evaluate randomization groups in terms of demographic, and initial clinical and kinematic characteristics, including PFP cause, delay since injury, side of paresis, previous drug treatments, and comorbidities. Quantitative data will be described using means (± SD) or medians (with IQR), depending on the normality of distributions, and qualitative data will be reported as numbers or percentages.

Changes in the Sunnybrook Composite score between D1 and D180 will be evaluated by Student’s *t* test. No interim analysis has been planned. The primary outcome analysis will be performed using Intention to treat (ITT) analysis. Comparative analysis of quantitative values at J90 and J180 – including primary and secondary outcomes—will be performed with Student’s *t* tests for independent samples or with Mann–Whitney non-parametric tests, depending on conditions. Changes in quantitative values from inclusion to each assessment visit (D90-D1, D180-D1) will be compared taking into account the baseline data in case of baseline intergroup differences despite randomization. Within-group changes will be tested through Student’s *t*-tests for matched data or Wilcoxon Rank sum tests, depending on the conditions. Multiple linear regression models will be tested to identify independent predictors associated with the changes in quantitative parameters over time, including longitudinal analysis of random effects (mixed models).

Comparative analysis of binary parameters will be run through Chi-square or Fisher’s exact tests, depending on conditions. Multivariable analysis will be generated through logistic regression models. Intra- and inter-rater reliability studies will use frequencies of agreement, intraclass correlation coefficients, Cohen and Fleiss kappa coefficients and coefficients of variation. Spearman or Pearson coefficients will be calculated between scores on the various scales, depending on conditions. Complementary analyses on the primary outcome and all analyses regarding secondary outcomes will be performed in both ITT and per protocol (PP) populations to describe the excluded patients of the PP population and to evaluate the robustness of the results.

All missing or invalid data will be systematically sought for verification in patient charts. In addition to the analysis performed on complete cases without missing data for the primary outcome, sensitivity analysis will be performed using various methods of replacement of missing data, including the LOCF method (Last Observation Carry Forward), the worst-case assumption, and multiple account assignment by chained equations (MICE). Participants who terminate the study prematurely will not be replaced. All analyzes will be performed with Stata software v14.1 (StataCorp, College Station, TX, USA).

### Safety

Assistance Publique—Hôpitaux de Paris (APHP), sponsor of this research, has contracted an insurance for the duration of the study, in compliance with the law on biomedical research, guaranteeing its own civil liability, as well as that of any investigator or staff involved in conducting the research.

## Discussion

To our knowledge, this study will be the first controlled study evaluating a facial motor strengthening program, through the strategy of Guided Self-rehabilitation Contracts, in the challenging treatment of chronic facial paresis. The study also represents uncommon involvement of a neurorehabilitation team, classically involved with central motor disorders, into the field of peripheral facial paresis. There are a number of arguments that support the plausibility that motor strengthening might help patients: (i) recover better emotional facial animation and (ii) reduce facial muscle overactivity.

### Why facial motor strengthening in chronic facial paresis?

The goal of a motor strengthening program for facial paresis is to provide for a fitter *peripheral* execution—through restaured facial nerve function—of the emotional command to facial muscles. The important study by Beurskens and Heymans, 2006 showed that re-intensification of motor work could achieve improvements in a chronic facial paresis population [62].

In terms of peripheral transmission of the emotional motor command, the phenomenon of activity-dependent plasticity of peripheral *synapses* has long been demonstrated by Klein and Kandel’s work [64]. As for *conduction* along peripheral axons, this is also a plastic phenomenon that acutely responds to intense work along the peripheral nerve [65] and studies also support chronic, training-induced, activity-dependent remyelination of peripheral neurons [63, 66]. The latter studies thus support the hypothesis that involving the facial nerve in chronic repeated work might improve conduction along its axons and thus improve the peripheral expression of emotional command.

In terms of muscle overactivity and excitability of the peripheral motoneurone, a number of studies show that increased descending muscle activation through strength training actually *reduces* motoneuronal overactivity, with increased post activation depression after long-term motor training [67]*.* In passing, the opposite is also true, as hyperexcitability of the peripheral motoneurone is known to be a subacute effect of *reduced* descending activation [68–70]. It is therefore unlikely that strength training programs might increase facial motoneuronal excitability; it is in fact possible to hypothesize that this rehabilitation technique might reduce facial motoneuronal excitability and thus facial muscle overactivity.

### Characteristics of the two rehabilitation groups

Several differences may be anticipated between the two rehabilitation treatments. Neither quality not quantity of rehabilitation have been imposed on the therapists involved in the control group, in order to best reflect real life. It is thus likely that frequency, techniques, duration of rehabilitation sessions will be highly heterogeneous in the control group. We have planned to collect these rehabilitation parameters (D90 and D180) in order to estimate the work carried out in that control group. Technically, in the rehabilitation techniques used in current practice, motor strengthening efforts are rarely recommended and used, particularly at maximal intensity as mentioned above, because of the concern or belief that this might trigger muscle overactivity. As a consequence, the amount of rehabilitative work classically performed typically avoids intensity and may thus be insufficient to exploit behavior-induced nervous system plasticity. From a psychological point of view, patient responsibilization and implication in the rehabilitation program to achieve higher work intensity levels constitute the essence of the Guided Self-rehabilitation Contract strategy. The patient has an active responsibility to accomplish the prescribed daily work and to notify it in the quantitative diary, in stark contrast with the common passive expectation of community therapy sessions in current practice.

Overall, this study should increase the level of knowledge on the effects of facial motor strengthening through Guided Self-rehabilitation Contracts in chronic stages. It will also improve the level of knowledge on facial motion quantification at rest and during voluntary and emotional facial contractions. The validation of a new clinical scale (the Créteil Scale) and of a wearable 3D motion analysis system of facial movements will help to better quantify PFP.

### Trial status

VISAGE trial has begun recruitment in March 2021. As of December 2022, 33/82 patients (40,2%) have been included. 

## Supplementary Information

**Additional file 1: Appendix A.** Study information and consent form.

**Additional file 2: Appendix B.** Rehabilitation booklet of the guided-self rehabilitation group.

**Additional file 3: Appendix C.** Rehabilitation booklet of the conventional therapy group.

## Data Availability

The dataset supporting this article is available at Henri Mondor University Hospitals, at the Clinical Research Unit, 1 rue Gustave Eiffel, 94,000 Créteil, France. ***Protocol amendments *** Any substantial modification of the protocol by the coordinating investigator must be transmitted to the sponsor for approval. After this approval, the sponsor must obtain a favorable opinion from the IRB prior to its implementation. Relevant parties (investigators, REC/IRBs, trial participants) will be contacted for communicating important protocol modifications by the coordinating investigator and/or the Clinical Research Unit by postal or electronic mail depending on the situation. ***Confidentiality of data*** Personal information about potential and enrolled participants will be collected, shared, and maintained in order to protect confidentiality before, during, and after the trial using a secured online database (eCRF) and an internal hospital server containing the password-protected database. ***Dissemination policy*** The sponsor AP-HP is the owner of the data. The data cannot be used or disclosed to a third party without its prior permission. Investigators and sponsor will communicate on trial results to participants, professionals and public through publications and oral and/or writing communications. The Coordinator investigator of each center including at least one patient will be eligible to authorship. Manuscripts related to the study results will be reviewed by a professional writer. The VISAGE project is registered on the APHP clinical research registry, free online access: https://www.aphp.fr/registre-des-essais-cliniques.

## Abbreviations

APHP: Assistance Publique—Hôpitaux de Paris
CNIL: Commission Nationale de l’Informatique et des Libertés
CPT: Conventional Physical Therapy
DRCI: Département de la Recherche Clinique et de l’Innovation
FACE: Facial Clinimetric Evaluation
GSC: Guided Self-rehabilitation Contract
HADS: Hospital Anxiety and Depression Scale
PFP: Peripheral Facial Paresis
SFGS: Sunnybrook Facial Grading System
