# Supplementary material for: Guided Self-rehabilitation Contract vs conventional therapy in chronic peripheral facial paresis: VISAGE, a multicenter randomized controlled trial
Source: BMC Neurol. 2023 Apr 10;23:148. doi: 10.1186/s12883-023-03096-8 (PMC10084642; doi:10.1186/s12883-023-03096-8)
Supplement: Supplementary file 1 — Additional file 1: Appendix A. Study information and consent form. [file 12883_2023_3096_MOESM1_ESM.pdf]

Impact clinique, cinématique et sur la qualité de vie de deux programmes de rééducation Dans la parésie faciale périphérique chronique : essai comparatif contrôlé randomisé *VISAGE*

Cette recherche est promue par l'Assistance Publique - Hôpitaux de Paris
Délégation à la Recherche Clinique et à l'Innovation
1 avenue Claude Vellefaux
75010 Paris

NOTE D'INFORMATION

Madame, Monsieur,

Le Docteur/ Le Professeur (barrer les mentions inutiles)…………………………………… (nom, prénom), exerçant à l'hôpital ……………………………………………, vous propose de participer à une recherche concernant votre état de santé.

Il est important de lire attentivement cette note avant de prendre votre décision. N'hésitez pas à demander des explications au médecin qui vous fournira cette note d'information.
Si vous décidez de participer à cette recherche, un consentement écrit vous sera demandé.

1) Quel est le but de cette recherche ?

Cette recherche dénommée *VISAGE* porte sur la récupération motrice de votre parésie faciale périphérique. Dans la littérature scientifique, il existe peu d'éléments sur la possibilité de récupération d'une parésie faciale périphérique au stade chronique par le biais d'une rééducation. Il n'existe pas de technique rééducative ayant clairement fait sa preuve ou supérieure à une autre, faute d'essais thérapeutiques de qualité suffisante.
L'objectif de cette étude est de comparer l'efficacité de deux programmes de rééducation de la parésie faciale périphérique chronique. Pour répondre à cette question, 82 personnes présentant une parésie faciale périphérique unilatérale chronique (depuis plus de 1 an le jour de l'inclusion) seront incluses dans 5 établissements de soins situés à Créteil, Paris et Versailles.

2) En quoi consiste la recherche ?

Cette recherche va permettre de comparer l'efficacité de deux programmes distincts de rééducation de la parésie faciale périphérique chronique. Pour cela, des évaluations seront réalisées, sur les plans clinique (évaluation de la motricité faciale), cinématique (étude en trois dimensions du mouvement facial) et de la qualité de vie (échelle de qualité de vie). Un tirage au sort (randomisation) déterminera initialement votre groupe de traitement parmi les deux groupes étudiés. Dans chacun des deux groupes un programme spécifique de rééducation faciale vous sera prescrit et devra être suivi tout au long des 6 mois de l'étude.
Une première étape de validation de la technique d'évaluation en trois dimensions du mouvement facial est prévue chez des patients exempts de parésie faciale périphérique chronique.

3) Quel est le calendrier de la recherche ?

La durée prévisionnelle de la recherche est d'environ 38 mois (env. 6 mois pour la première étape et 32 mois pour la deuxième étape pour laquelle vous êtes sollicité(e)) et votre participation sera au maximum de 8 mois en fonction de la programmation des visites.

Votre médecin vous a remis cette note d'information et informé de l'objectif et du déroulement de cette recherche au cours d'une consultation. Après avoir reçu toutes les explications et posé les questions que vous souhaitez sur cette recherche, vous bénéficiez d'un délai de réflexion suffisant pour décider de votre participation ou non à cette étude.

Ce document est la propriété de la DRCI - APHP. Toute reproduction est formellement interdite.
version 6.0 du 28/05/2019

A la visite d'inclusion, après la signature de votre consentement et la vérification des critères de sélection, vous serez randomisé(e) dans un des deux groupes de traitement. Celui-ci pourra vous être précisé brièvement (auto rééducation guidée par un thérapeute ou rééducation de ville) mais il vous sera véritablement expliqué et décrit à la première visite qui aura lieu à l'hôpital Mondor (J0). Ce jour-là une liste de thérapeutes de ville spécialisés dans la rééducation des parésies faciales vous sera donné pour faciliter votre prise en charge. Vous ne débuterez pas votre traitement rééducatif avant J0. Votre médecin vous donner un carnet-patient personnalisé qu'il faudra rapporter à chaque visite.

La 1ère visite d'évaluation ainsi que les deux autres visites auront lieu exclusivement dans le service de Rééducation Neurolocomotrice de l'hôpital Henri Mondor de Créteil : vous serez contacté par téléphone pour programmer ces **3 demi-journées d'évaluation**.

La première demi-journée d'évaluation aura lieu 30 jours après la visite d'inclusion et correspondra à la visite J0. Votre programme de rééducation ne commencera lui-même qu'à la suite de cette visite J0.

Les deux autres demi-journées d'évaluation auront lieu à 3 et 6 mois (J90 et J180).

A chaque demi-journée d'évaluation, deux consultations distinctes auront lieu :
- une consultation d'évaluation avec un médecin qui ne connaitra pas votre groupe de traitement : durant celle-ci, deux échelles cliniques dont une sera vidéo-enregistrée (Echelle de Créteil) seront réalisées. Le vidéo-enregistrement permettra d'évaluer la fiabilité de l'échelle grâce à sa relecture par des évaluateurs indépendants ;
- une consultation de rééducation avec un médecin qui connaitra votre groupe de traitement : durant celle-ci, le programme de rééducation vous sera expliqué et votre compliance au traitement sera évaluée.

Aux 3 visites d'évaluation, vous devrez répondre à 4 auto-questionnaires.

Entre les 3 demi-journées d'évaluation, vous recevrez 4 appels téléphoniques par le médecin de rééducation pour faire le point sur votre programme de rééducation. Votre programme de rééducation durera lui-même 6 mois, il vous aura été prescrit à la visite d'inclusion en fonction de votre groupe de traitement.

A la 1ère et 3ème visite d'évaluation (avant/après traitement), un enregistrement 3D des mouvements faciaux sera réalisé à l'aide d'un casque portatif muni de 4 caméras. Des marqueurs seront stratégiquement posés sur votre visage et permettront de suivre les mouvements de votre visage. Le temps de pose des marqueurs sera de 20 minutes et le temps d'enregistrement des mouvements faciaux de 15 minutes. L'enregistrement 3D ne sera pas réalisé à la 2ème visite d'évaluation.

Voici un tableau récapitulant le calendrier de la recherche VISAGE :

	**Inclusion**	**Visite 1**	**Appel téléphone 1**	**Appel téléphone 2**	**Visite 2**	**Appel téléphone 3**	**Appel téléphone 4**	**Visite 3**
Lieu	Centres investigateurs où vous consultez habituellement	Hôpital Mondor			Hôpital Mondor			Hôpital Mondor
Jour	J-30	J0	J30	J60	J90	J120	J150	J180
Durée totale	**30 min**	**3h**	**10 min**	**10 min**	**2h**	**10 min**	**10 min**	**3h**
Consultation de rééducation		x			x			x
Consultation d'évaluation		x			x			x
Analyse 3D		x						x
Auto-questionnaires		x			x			x

Ce document est la propriété de la DRCI - APHP. Toute reproduction est formellement interdite.
version 6.0 du 28/05/2019

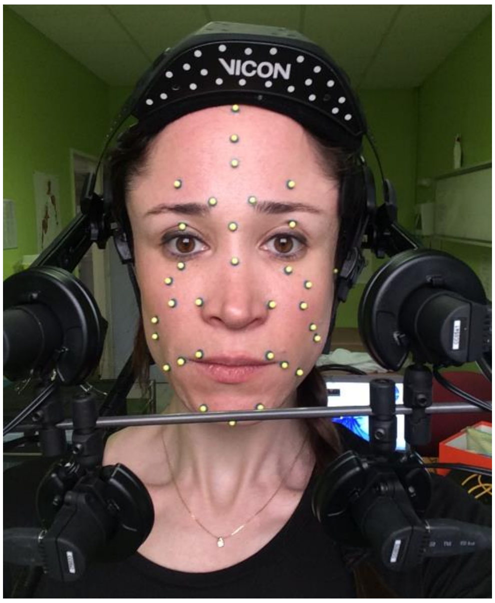

Dispositif d'analyse 3D des mouvements du visage

4) Quels sont les bénéfices liés à votre participation

Grâce à votre participation, vous contribuerez à évaluer et comparer deux programmes de rééducation faciale et probablement à prouver qu'une récupération motrice est possible plusieurs années après le début des symptômes, stade où la plupart des patients ne sont plus pris en charge par le milieu médical et/ou paramédical.
Vous permettrez également de valider de nouveaux outils d'évaluation de votre parésie faciale périphérique (échelle clinique de motricité faciale, évaluation des mouvements de la face en trois dimensions, échelle de qualité de vie) permettant un suivi fiable et quantifié de votre pathologie. Ces mêmes outils permettront de suivre l'efficacité d'autres thérapeutiques ayant pu vous être proposées (autres programmes de rééducation, chirurgie faciale, injections de toxine botulique).

5) Quels sont les traitements autorisés et non autorisés ?

Durant votre participation à l'étude, vous ne devrez pas recevoir d'injection de toxine botulique, d'acide hyaluronique, ni réaliser de lipofilling ou encore de traitement par laser au niveau du visage. Vous ne devez pas non plus subir d'intervention chirurgicale au niveau du visage. Ces interventions pourront par contre être réalisées dès la fin de votre participation à l'étude.
Tous les autres traitements seront *a priori* autorisés mais il sera nécessaire d'en informer le médecin le jour de la visite d'inclusion.

6) Quels sont les risques et les contraintes prévisibles ajoutés par la recherche?

Les risques d'une participation à un programme de rééducation faciale seraient de présenter une fatigue musculaire transitoire au niveau du visage ou de développer une hyperactivité de certains groupes musculaires faciaux. Si des effets secondaires étaient amenés à survenir pendant votre participation, vous serez pris en charge immédiatement dans le service par le biais d'une consultation et bénéficierez d'un suivi rapproché. Dans le cadre du protocole, les visites d'évaluation et les appels téléphoniques mensuels permettent de garantir une surveillance optimale de votre état de santé.

Si vous acceptez de participer à cette recherche, vous devrez respecter les contraintes suivantes :

- Vous présentez aux rendez-vous à l'hôpital Henri-Mondor (Créteil); en cas d'impossibilité, il vous sera demandé de prévenir le médecin le plus rapidement possible pour un nouveau rendez-vous.
- Effectuer votre programme de rééducation faciale durant 6 mois soit en ville soit à domicile.
- Informer le médecin de la recherche de l'utilisation de tout autre traitement ainsi que de tout événement survenant pendant la recherche (hospitalisation pour toute autre raison, abandon du traitement, etc).
- Tenir votre recueil des séances de rééducation sur un carnet qui vous sera remis au début de l'étude et rapporter le carnet rempli à chaque demi-journée d'évaluation.

Ce document est la propriété de la DRCI - APHP. Toute reproduction est formellement interdite.

- Ne pas recevoir d'injection de toxine botulique ou de chirurgie durant les 8 mois de l'étude.
- Etre d'accord de vous raser ou de ne pas vous maquillez pour les visites de la recherche (inclusion et les 3 demi-journées d'évaluation)
- Ne pas prendre part à un autre projet de recherche sans l'accord du médecin investigateur.
- Etre affilié(e) à un régime de sécurité sociale ou être bénéficiaire d'un tel régime.

Vous recevrez une indemnité d'un montant de 200 euros pour couvrir vos 3 déplacements correspondant aux 3 visites d'évaluation si vous vous présentez à la totalité des évaluations (3 visites complètes).

7) Quels sont les éventuelles alternatives médicales ?

Au stade chronique d'une parésie faciale, la majorité des patients ne bénéficie plus d'aucune prise en charge rééducative alors que certains travaux scientifiques ainsi que notre pratique clinique montrent qu'une récupération motrice est possible même après plusieurs années d'évolution. Il n'existe donc pas d'alternative médicale dans le cadre de cette étude.

8) Quelles sont les modalités de prise en charge médicale à la fin de votre participation ?

A la fin de votre participation à l'étude, vous pourrez être suivi dans un des 5 centres participant à l'étude. Lorsque l'étude sera totalement terminée, le médecin qui vous suit pourra choisir avec vous de poursuivre la rééducation ainsi le type de programme qui semble le plus adapté.

Votre médecin pourra décider à tout moment de l'arrêt de votre participation ; il vous en expliquera les raisons.

9) Si vous participez, comment vont être traitées les données recueillies pour la recherche ?

Dans le cadre de la recherche à laquelle l'AP-HP vous propose de participer, un traitement de vos données personnelles va être mis en œuvre pour permettre d'en analyser les résultats.

Ce traitement est nécessaire à la réalisation de la recherche qui répond à la mission d'intérêt public dont est investie l'AP-HP en tant qu'établissement public de santé hospitalo-universitaire.

A cette fin, les données médicales vous concernant et les données relatives à vos habitudes de vie, seront transmises au Promoteur ou aux personnes ou partenaires agissant pour son compte, en France. Ces données seront identifiées par un numéro d'enregistrement. Ces données pourront également, dans des conditions assurant leur confidentialité, être transmises aux autorités de santé françaises.

Les données médicales vous concernant pouvant documenter un dossier auprès des autorités compétentes pourront être transmises à un industriel afin qu'un plus grand nombre de patients puissent bénéficier des résultats de la recherche. Cette transmission sera faite dans les conditions assurant leur confidentialité.

Vos données pourront être utilisées pour des recherches ultérieures ou des analyses complémentaires à la présente recherche, en collaboration avec des partenaires privés ou publics, en France ou à l'étranger, dans des conditions assurant leur confidentialité et le même niveau de protection que la législation européenne.
Vous pouvez vous opposer à tout moment à l'utilisation ultérieure de vos données auprès du médecin qui vous suit dans le cadre de cette recherche
Vos données ne seront conservées que pour une durée strictement nécessaire et proportionnée à la finalité de la recherche. Elles seront conservées dans les systèmes d'information du responsable de traitement jusqu'à deux ans après la dernière publication des résultats de la recherche.

Le fichier informatique utilisé pour cette recherche est mis en œuvre conformément à la règlementation française (loi Informatique et Libertés modifiée) et européenne (au Règlement Général sur la Protection des Données -RGPD), Vous disposez d'un droit d'accès, de rectification, de limitation et d'opposition au traitement des données couvertes par le secret professionnel utilisées dans le cadre de cette recherche. Ces droits s'exercent auprès du médecin en charge de la recherche qui seul connaît votre identité (identifié en première page du présent document).

Ce document est la propriété de la DRCI - APHP. Toute reproduction est formellement interdite.
version 6.0 du 28/05/2019

Si vous décidez d'arrêter de participer à la recherche, les données recueillies précédemment à cet arrêt seront utilisées conformément à la réglementation, et exclusivement pour les objectifs de cette recherche. En effet, leur effacement serait susceptible de compromettre la validité des résultats de la recherche. Dans ce cas, vos données ne seront absolument pas utilisées ultérieurement ou pour une autre recherche.

En cas de difficultés dans l'exercice de vos droits, vous pouvez saisir le Délégué à la Protection des données de l'AP-HP à l'adresse suivante : protection.donnees.dsi@aphp.fr, qui pourra notamment vous expliquer les voies de recours dont vous disposez auprès de la CNIL. Vous pouvez également exercer votre droit à réclamation directement auprès de la CNIL (pour plus d'informations à ce sujet, rendez-vous sur le site www.cnil.fr).

11) Comment cette recherche est-elle encadrée ?

L'AP-HP a pris toutes les mesures pour mener cette recherche conformément aux dispositions du Code de la Santé Publique applicables aux recherches impliquant la personne humaine. L'AP-HP a souscrit une assurance (N° d'adhésion : 0100518814033-160021-10998) garantissant sa responsabilité civile et celle de tout intervenant auprès de la compagnie HDI–GERLING par l'intermédiaire de BIOMEDICINSURE dont l'adresse est Parc d'Innovation Bretagne Sud C.P.142 56038 Vannes Cedex. L'AP-HP a obtenu l'avis favorable du Comité de Protection des Personnes Est-III pour cette recherche lors de la séance du 02/10/2018.

12) Quels sont vos droits ?

Votre participation à cette recherche est entièrement libre et volontaire. Votre décision n'entraînera aucun préjudice sur la qualité des soins et des traitements que vous êtes en droit d'attendre.

Vous pourrez tout au long de la recherche demander des informations concernant votre santé ainsi que des explications sur le déroulement de la recherche au médecin qui vous suit.

Vous pouvez vous retirer à tout moment de la recherche sans justification, sans conséquence sur la suite de votre traitement ni la qualité des soins qui vous seront fournis et sans conséquence sur la relation avec votre médecin. A l'issue de ce retrait, vous pourrez être suivi par la même équipe médicale. Dans ce cas, les données collectées jusqu'au retrait seront utilisées pour l'analyse des résultats de la recherche.

Votre dossier médical restera confidentiel et ne pourra être consulté que sous la responsabilité du médecin s'occupant de votre traitement ainsi que par les autorités de santé et par des personnes dûment mandatées par l'AP-HP pour la recherche et soumises au secret professionnel.

A l'issue de la recherche et après analyse des données relatives à cette recherche, vous pourrez être informé(e) des résultats globaux en le demandant au médecin qui vous suit dans le cadre de cette recherche.

Vous pouvez également accéder directement ou par l'intermédiaire d'un médecin de votre choix à l'ensemble de vos données médicales en application des dispositions de l'article L 1111-7 du Code de la Santé Publique.

Après avoir lu toutes ces informations, discuté tous les aspects avec votre médecin et après avoir bénéficié d'un temps de réflexion, si vous acceptez de participer à la recherche vous devrez signer et dater le formulaire de consentement éclairé se trouvant à la fin de ce document.

Ce document est la propriété de la DRCI - APHP. Toute reproduction est formellement interdite.
version 6.0 du 28/05/2019

ASSISTANCE PUBLIQUE HÔPITAUX DE PARIS

FORMULAIRE DE CONSENTEMENT

Je soussigné(e), Mme, M. *[rayer les mentions inutiles]* (*nom, prénom*)……………………………………………………..
accepte librement de participer à la recherche intitulée « Impact clinique, cinématique et sur la qualité de vie de deux programmes de rééducation dans la parésie faciale périphérique chronique : essai comparatif contrôlé randomisé » organisée par l'Assistance Publique - Hôpitaux de Paris et qui m'est proposée par le Docteur (*nom, prénom, téléphone*) …………………………………………………………………………….…, médecin dans cette recherche.

- J'ai pris connaissance de la note d'information version 3.0 du 30/01/2020 de 5 pages m'expliquant l'objectif de cette recherche, la façon dont elle va être réalisée et ce que ma participation va impliquer,
- je conserverai un exemplaire de la note d'information et du consentement,
- j'ai reçu des réponses adaptées à toutes mes questions,
- j'ai disposé d'un temps suffisant pour prendre ma décision,
- j'ai compris que ma participation est libre et que je pourrai interrompre ma participation à tout moment, sans encourir la moindre responsabilité et préjudice pour la qualité des soins qui me seront prodigués.
- j'ai été informé que les données recueillies dans le cadre de la recherche peuvent être réutilisées pour des recherches ultérieures, et que je pouvais m'y opposer à tout moment. Il pourra s'agir notamment de l'utilisation des données d'analyse 3D de mes mouvements faciaux dans le cadre d'autres études.
- je suis conscient(e) que ma participation pourra aussi être interrompue par le médecin si besoin, il m'en expliquera les raisons,
- j'ai compris que pour pouvoir participer à cette recherche je dois être affilié(e) à un régime de sécurité sociale ou bénéficiaire d'un tel régime. Je confirme que c'est le cas,
- j'ai bien été informé(e) que ma participation à cette recherche durera 8 mois maximum, et que cela implique que je ne pourrai pas envisager de participer à une autre recherche avant la fin de l'étude sans en informer le médecin qui me suit pour la recherche,
- j'ai été informé(e) que les enregistrements vidéographiques identifiants et cinématiques réalisés dans le cadre de cette recherche, seront conservés et utilisés ultérieurement à des fins de recherche portant sur l'analyse du mouvement facial,
- mon consentement ne décharge en rien le médecin qui me suit dans le cadre de la recherche ni l'AP-HP de l'ensemble de leurs responsabilités et je conserve tous mes droits garantis par la loi.

**Signature de la personne participant à la recherche**		**Signature du médecin**	
Nom Prénom :		Nom Prénom :	
Date :	Signature :	Date :	Signature :

Ce document est à réaliser en 3 exemplaires, un exemplaire doit être conservé 15 ans par l'investigateur, le deuxième remis à la personne donnant son consentement et le troisième transmis à l'AP-HP sous enveloppe scellée à la fin de la recherche.

Ce document est la propriété de la DRCI - APHP. Toute reproduction est formellement interdite.
version 6.0 du 28/05/2019

FORMULAIRE DE CONSENTEMENT

Je soussigné(e), Mme, M. *[rayer les mentions inutiles]* (*nom, prénom*)…………………………………………………….... **accepte librement de participer à la recherche intitulée** « Impact clinique, cinématique et sur la qualité de vie de deux programmes de rééducation dans la parésie faciale périphérique chronique : essai comparatif contrôlé randomisé » organisée par l'Assistance Publique - Hôpitaux de Paris et qui m'est proposée par le Docteur (*nom, prénom, téléphone*) ………………………………………………………………………….., médecin dans cette recherche, **et que :**

Les vidéographies réalisées dans le cadre du protocole puissent être utilisées pour réaliser des recherches scientifiques ultérieures, des enseignements et/ou présentations en congrès.

**Signature de la personne participant à la recherche**		**Signature du médecin**	
Nom Prénom :		Nom Prénom :	
Date :	Signature :	Date :	Signature :

Ce document est à réaliser en 3 exemplaires, un exemplaire doit être conservé 15 ans par l'investigateur, le deuxième remis à la personne donnant son consentement et le troisième transmis à l'AP-HP sous enveloppe scellée à la fin de la recherche.

Ce document est la propriété de la DRCI - APHP. Toute reproduction est formellement interdite.
version 6.0 du 28/05/2019
