# Supplementary material for: Guided Self-rehabilitation Contract vs conventional therapy in chronic peripheral facial paresis: VISAGE, a multicenter randomized controlled trial
Source: BMC Neurol. 2023 Apr 10;23:148. doi: 10.1186/s12883-023-03096-8 (PMC10084642; doi:10.1186/s12883-023-03096-8)
Supplement: Supplementary file 2 — Additional file 2: Appendix B. Rehabilitation booklet of the guided-self rehabilitation group. [file 12883_2023_3096_MOESM2_ESM.pdf]

Carnet de suivi

VISAGE

Groupe Autorééducation

Effet clinique, cinématique et sur la qualité de vie de deux programmes de rééducation dans la parésie faciale périphérique chronique

Merci de compléter ce carnet et de le rapporter à votre médecin à chaque consultation.

***Les coordonnées de votre médecin figurent à la fin de ce carnet*.**

Votre identifiant dans l'étude : **I__I__I__I - I__I__I__I__I - I__I__I**
(n° de centre, n° d'inclusion, Initiale du nom, Initiale du prénom)

Nom de l'investigateur:

Adresse:

Préambule

Madame, Monsieur,

L'objectif de l'étude à laquelle vous avez accepté de participer est de comparer deux programmes de rééducation dans la récupération motrice d'une parésie faciale périphérique unilatérale au stade chronique.

Lors de la visite d'inclusion, vous avez été tiré au sort dans le groupe Autorééducation. Ce groupe implique la réalisation d'un programme défini d'entrainement moteur intensif de certains groupes musculaires faciaux en séries fatigantes de contractions maximales. Il repose également sur la tenue quotidienne rigoureuse d'un registre d'exercices dans ce carnet. La poursuite d'une autre prise en charge rééducative n'est pas interdite mais nécessite que vous reportiez la durée et la fréquence des séances ainsi que la réalisation d'un autre travail éventuel à domicile.

Il est important d'apporter ce carnet à chaque visite.

Consignes importantes

→ Il est nécessaire que les hommes effectuent un rasage la veille au soir ou le matin des 3 demi-journées d'évaluation pour ne pas gêner les évaluations.

→Il est nécessaire que les femmes ne se maquillent pas ou ne portent pas de crème le matin des 3 demi-journées d'évaluation pour ne pas gêner les évaluations.

→Le jour des 3 visites (J0, J90 et J180), il est nécessaire de porter un haut de couleur noire et de retirer tous les bijoux au niveau du visage, du cou et des oreilles.

→ Il est important de réaliser votre programme d'exercices <u>quotidiennement</u> et de noter rigoureusement sur le <u>registre chaque jour</u> vos exercices effectués.

Rappel des traitements interdits durant l'étude

Durant l'étude, certains traitements sont susceptibles de compromettre les évaluations.

Il est important de ne pas avoir recours aux traitements suivants durant votre participation à l'étude :

- Injections de toxine botulique au visage
- Injections d'acide hyaluronique au visage
- Injections de graisse au visage
- Laser au visage
- Chirurgie au visage

Visite d'inclusion : J-30

- Elle a lieu 30 jours avant le début des évaluations
- Durée : 30 minutes
- Dans un des 5 centres d'inclusion de l'étude
- Déroulement :
  - Explication de l'étude VISAGE
  - Réponses aux questions éventuelles
  - Vérification des critères d'inclusion et de non-inclusion
  - Remise de la note d'information et signature du consentement
  - Inclusion et randomisation dans l'étude
  - Remise de votre carnet-patient à ramener à chaque journée d'évaluation : J0, J90 et J180
- La date de la première évaluation, c'est-à-dire J0, vous sera communiquée dans les jours suivants par un appel téléphonique donné par le service de Rééducation Neurolocomotrice de l'hôpital Henri Mondor de Créteil (centre évaluateur de l'étude).

Appels téléphoniques

- Durée : 5 minutes par appel
- Vous recevrez 4 appels téléphoniques brefs réalisés par le Dr Gault-Colas, médecin rééducateur affilié à l'étude
- Les appels seront programmés selon le calendrier suivant :
  - J30
  - J60
  - J120
  - J150

- Ils permettront de vérifier la poursuite de votre rééducation
- Le jour et l'heure de chaque appel sera décidé conjointement entre le Dr Gault-Colas et vous.

Evaluations : J0 – J90 – J180

Ces 3 évaluations auront exclusivement lieu à l'adresse suivante :

Service de Rééducation Neurolocomotrice de l'hôpital Henri Mondor
Rez de chaussée bas – 51 av du M^{al} de Lattre de Tassigny – 94010 Créteil

1ère évaluation - J0 :

- Durée : 2h30
- Consultation d'évaluation : échelles cliniques, évaluation 3D, auto-questionnaires
- Consultation de rééducation : prescription et explication du programme de rééducation faciale

2ème évaluation - J90 :

- Durée : 1h30
- **Apporter votre registre d'exercices rempli au sein de votre carnet-patient**
- Consultation d'évaluation : échelles cliniques, auto-questionnaires
- Consultation de rééducation : vérification du registre et de la bonne réalisation de la rééducation

3ème évaluation - J180 :

- Durée : 2h30
- **Apporter votre registre d'exercices rempli et votre carnet-patient**
- Consultation d'évaluation : échelles cliniques, évaluation 3D, auto-questionnaires
- Consultation de rééducation : vérification du registre d'exercices, de la bonne réalisation de la rééducation

Agenda de votre participation à l'étude

	**J-30 Inclusion**	**J0 Evaluation 1**	J30 Appel 1	J60 Appel 2	**J90 Evaluation 2**	J120 Appel 3	J150 Appel 4	**J180 Evaluation 3**
Date								
Note d'information	X							
Signature du consentement	X							
Vérification critères de sélection	X							
Randomisation	X							
Prescription de l'Autorééducation		X						
Echelles cliniques	X	X			X			X
Auto-questionnaires		X			X			X
Analyse 3D		X						X
Recueil effets indésirables					X			X
Appels téléphoniques			X	X		X	X	

Renforcement moteur
Muscle Frontal

Objectifs :

Améliorer la symétrie du front au repos
Améliorer la mobilité du front pendant la conversation

Outil recommandé : un miroir

Position de départ Assis, devant le miroir

Mouvement Relever les **deux** sourcils le plus fort possible **pendant 3 secondes**. Faîtes une pause de 1 seconde avant de recommencer le mouvement suivant.

Exercice Chaque série de mouvements s'effectue jusqu'à une sensation de fatigue franche obligeant à l'arrêt. La fatigue doit être obtenue en 20-40 répétitions. S'il n'y a toujours pas de sensation de fatigue obligeant à l'arrêt à 40 répétitions, arrêtez-vous.

- **Effectuer 3 séries par jour**
- **Noter sur le registre le nombre de mouvements de relever de sourcils effectués à chaque série.**

Position de départ

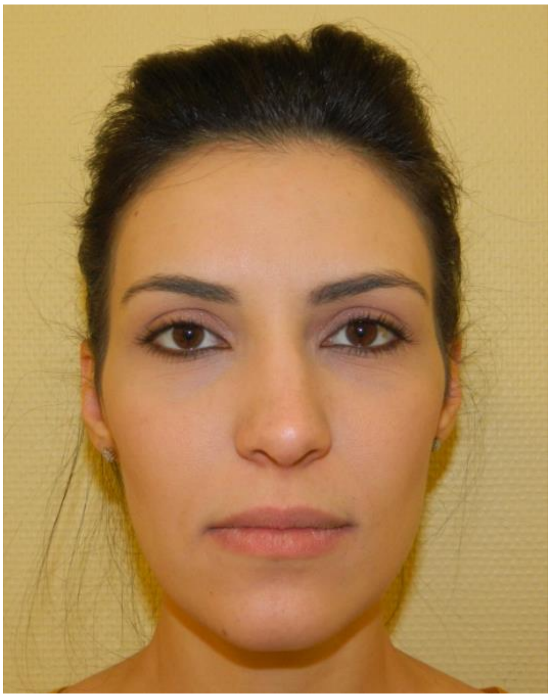

Mouvement

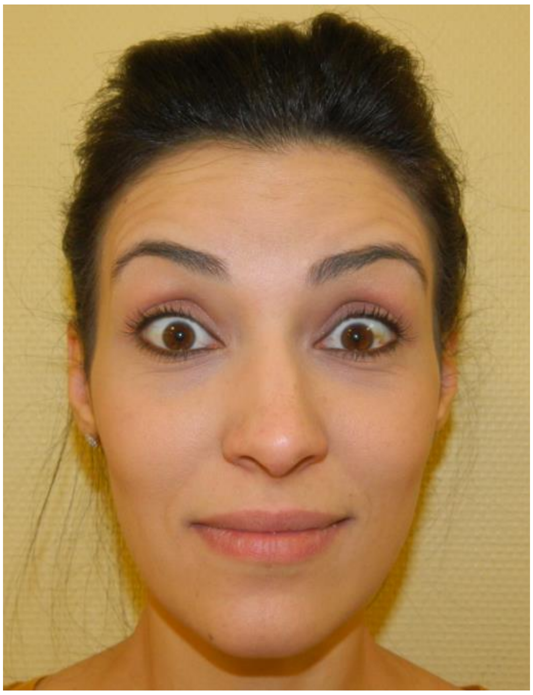

Renforcement moteur
Muscle Orbiculaires des paupières

Objectifs :

Améliorer la symétrie et la fermeture de votre œil au repos Et en activité

Outil recommandé : un miroir

Position de départ Assis, devant le miroir

Mouvement Fermer les **deux** yeux le plus fort possible **pendant 3 secondes**. Faîtes une pause de 1 seconde avant de recommencer le mouvement suivant.

Exercice Chaque série de mouvements s'effectue jusqu'à une sensation de fatigue franche obligeant à l'arrêt. La fatigue doit être obtenue en 20-40 répétitions. S'il n'y a toujours pas de sensation de fatigue obligeant à l'arrêt à 40 répétitions, il faut recommencer avec un poids plus lourd.

- **Effectuer 3 séries par jour**
- **Noter sur le registre le nombre de mouvements de fermeture des yeux effectués à chaque série.**

Position de départ

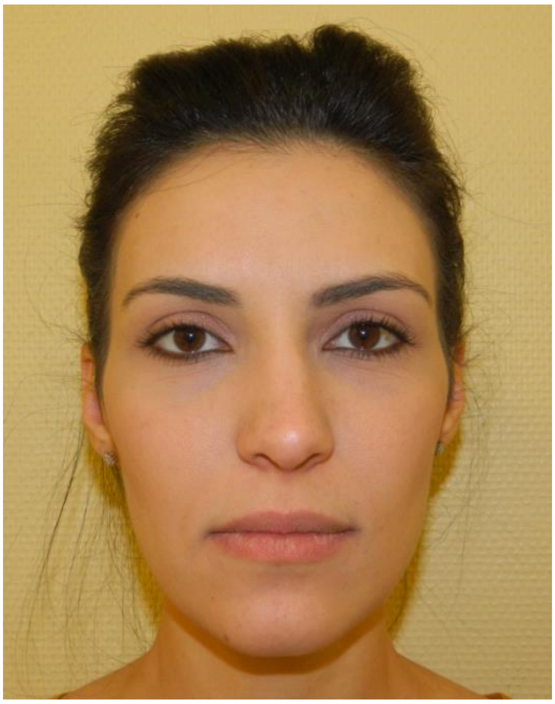

Mouvement

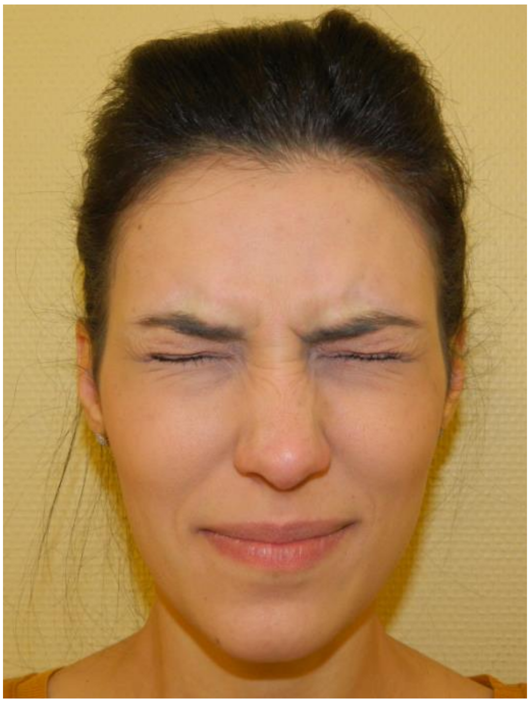

Renforcement moteur
Zygomatiques

Objectifs :

Améliorer la symétrie de votre bouche au repos, pendant la conversation et lors du sourire

Outil recommandé : un miroir

Position de départ Assis, devant le miroir

Mouvement Souriez en montrant les dents le plus fort possible **pendant 3 secondes**. Faîtes une pause de 1 seconde avant de recommencer le mouvement suivant.

Exercice Chaque série de mouvements s'effectue jusqu'à une sensation de fatigue franche obligeant à l'arrêt. La fatigue doit être obtenue en 20-40 répétitions. S'il n'y a toujours pas de sensation de fatigue obligeant à l'arrêt à 40 répétitions, arrêtez.

- **Effectuer 3 séries par jour**
- **Noter sur le registre le nombre de mouvements de sourires effectués à chaque série.**

Position de départ

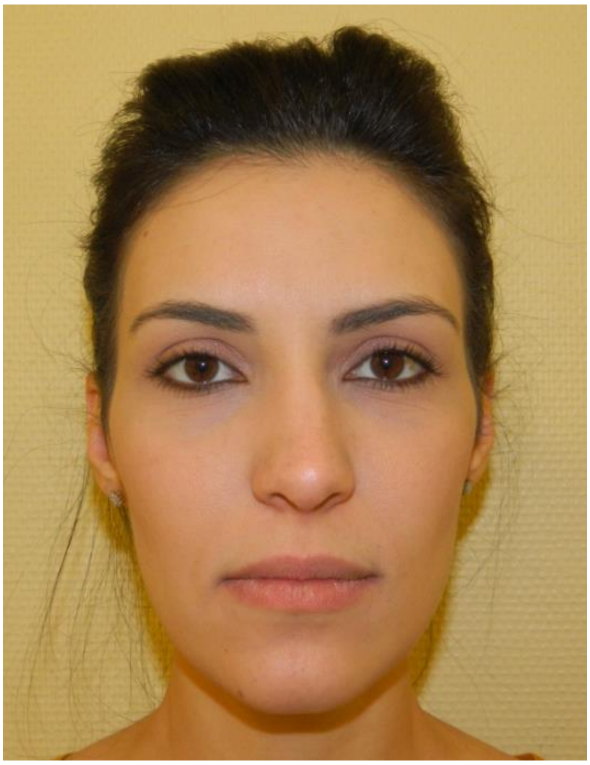

Mouvement

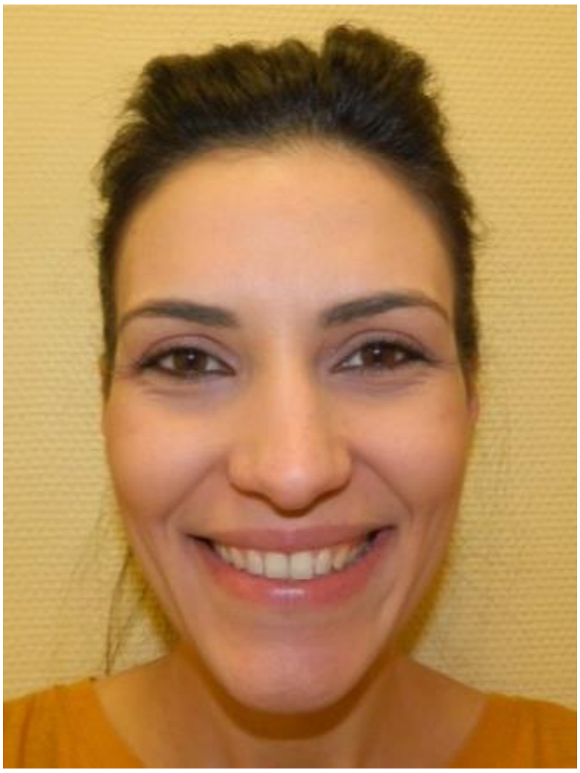

Contacts

En cas d'impossibilité de vous rendre à une des évaluations, merci de prévenir le service aux coordonnées téléphoniques suivantes :

- Accueil du service – Hôpital Mondor : 01 49 81 24 97 ;
- Secrétariat médical – Mme COOLS – Hôpital Mondor : 01 49 81 24 98 ;
- Secrétariat médical – Mme BLONDEL – Hôpital Chenevier : 01 49 81 30 61.

En cas de nécessité ou de problème médical, vous pouvez joindre le Docteur Dr GAULT-COLAS, médecin de rééducation de l'étude, au courant de votre groupe de traitement, aux coordonnées suivantes :

Service de Rééducation Neurolocomotrice - Hôpital Henri Mondor de Créteil

Secrétariat téléphonique : 01 49 81 24 98 - 01 49 81 24 97 - 01 49 81 30 61

En cas d'absolue nécessité et si les numéros ci-dessus ne fonctionnent pas, vous pouvez joindre le Dr BAUDE, médecin coordinateur principal de l'étude, aux coordonnées suivantes :

Service de Rééducation Neurolocomotrice - Hôpital Henri Mondor de Créteil

Secrétariat téléphonique : 01 49 81 24 98 - 01 49 81 24 97 - 01 49 81 30 61

Adresse mail : marjolaine.baude@aphp.fr

Ordonnance d’Autorééducation

Pour les 3 muscles indiqués ci-dessous, effectuez si possible devant miroir des séries de contractions maximales pendant 3 secondes, avec une très courte pause de moins d’une seconde entre deux contractions successives. Chaque série de contractions s’effectue jusqu’à une sensation de fatigue franche obligeant à l’arrêt. N**oter sur le registre le nombre de mouvements effectués à chaque série.**

1) Frontal :

3 séries fatigantes par jour

-Relevez les deux sourcils le plus fort possible pendant 3 secondes,

-Relâchez une seconde, puis recommencez.

Notez sur le registre le nombre de mouvements Effectués à chaque série.

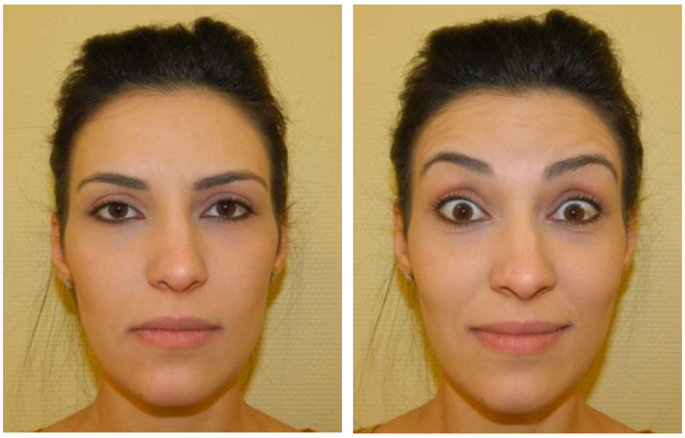

2) Orbiculaire des paupières :

3 séries fatigantes/jour

-Fermez les deux yeux le plus fort possible pendant 3 secondes,

-Relâchez une seconde, puis recommencez.

Notez sur le registre le nombre de mouvements Effectués à chaque série.

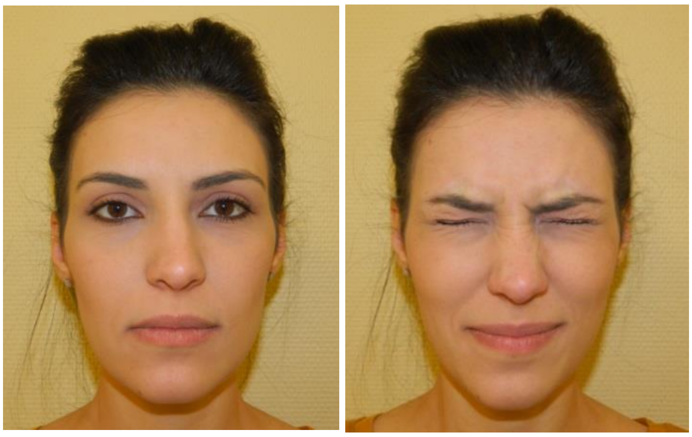

3) Zygomatiques :

3 séries fatigantes/jour

-Souriez le plus fort possible bouche ouverte pendant 3 secondes,

-Relâchez une seconde, puis recommencez.

Notez sur le registre le nombre de mouvements Effectués à chaque série.

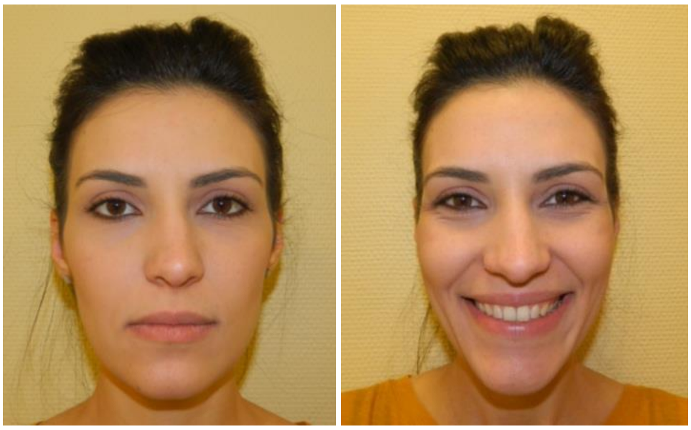

Tampon et signature :

Registre d'exercices

DATE	Frontal<br>*Relevez fort les sourcils*			Orbiculaire paupières<br>*Fermez fort les yeux*			Zygomatiques<br>*Souriez fort*		
	Nb de mouvements par série jusqu'à la fatigue			Nb de mouvements par série jusqu'à la fatigue			Nb de mouvements par série jusqu'à la fatigue		
	Série 1	Série 2	Série 3	Série 1	Série 2	Série 3	Série 1	Série 2	Série 3
J1									
J2									
J3									
J4									
J5									
J6									
J7									
J8									
J9									
J10									
J11									
J12									
J13									
J14									
J15									
J16									
J17									
J18									
J19									
J20									
J21									
J22									
J23									
J24									
J25									
J26									
J27									
J28									
J29									
J30									

Registre d’exercices

DATE	Frontal<br>*Relevez fort les sourcils*			Orbiculaire paupières<br>*Fermez fort les yeux*			Zygomatiques<br>*Souriez fort*		
	Nb de mouvements par série jusqu’à la fatigue			Nb de mouvements par série jusqu’à la fatigue			Nb de mouvements par série jusqu’à la fatigue		
	Série 1	Série 2	Série 3	Série 1	Série 2	Série 3	Série 1	Série 2	Série 3
J31									
J32									
J33									
J34									
J35									
J36									
J37									
J38									
J39									
J40									
J41									
J42									
J43									
J44									
J45									
J46									
J47									
J48									
J49									
J50									
J51									
J52									
J53									
J54									
J55									
J56									
J57									
J58									
J59									
J60									

Registre d’exercices

DATE	**Frontal** *Relevez fort les sourcils*			**Orbiculaire paupières** *Fermez fort les yeux*			**Zygomatiques** *Souriez fort*		
	Nb de mouvements par série jusqu’à la fatigue			Nb de mouvements par série jusqu’à la fatigue			Nb de mouvements par série jusqu’à la fatigue		
	Série 1	Série 2	Série 3	Série 1	Série 2	Série 3	Série 1	Série 2	Série 3
J61									
J62									
J63									
J64									
J65									
J66									
J67									
J68									
J69									
J70									
J71									
J72									
J73									
J74									
J75									
J76									
J77									
J78									
J79									
J80									
J81									
J82									
J83									
J84									
J85									
J86									
J87									
J88									
J89									
J90									

Registre d'exercices

DATE	**Frontal** *Relevez fort les sourcils*			**Orbiculaire paupières** *Fermez fort les yeux*			**Zygomatiques** *Souriez fort*		
	Nb de mouvements par série jusqu'à la fatigue			Nb de mouvements par série jusqu'à la fatigue			Nb de mouvements par série jusqu'à la fatigue		
	Série 1	Série 2	Série 3	Série 1	Série 2	Série 3	Série 1	Série 2	Série 3
J91									
J92									
J93									
J94									
J95									
J96									
J97									
J98									
J99									
J100									
J101									
J102									
J103									
J104									
J105									
J106									
J107									
J108									
J109									
J110									
J111									
J112									
J113									
J114									
J115									
J116									
J117									
J118									
J119									
J120									

Registre d’exercices

DATE	**Frontal** *Relevez fort les sourcils*			**Orbiculaire paupières** *Fermez fort les yeux*			**Zygomatiques** *Souriez fort*		
	Nb de mouvements par série jusqu’à la fatigue			Nb de mouvements par série jusqu’à la fatigue			Nb de mouvements par série jusqu’à la fatigue		
	Série 1	Série 2	Série 3	Série 1	Série 2	Série 3	Série 1	Série 2	Série 3
J121									
J122									
J123									
J124									
J125									
J126									
J127									
J128									
J129									
J130									
J131									
J132									
J133									
J134									
J135									
J136									
J137									
J138									
J139									
J140									
J141									
J142									
J143									
J144									
J145									
J146									
J147									
J148									
J149									
J150									

Registre d'exercices

DATE	**Frontal** ***Relevez fort les sourcils***			**Orbiculaire paupières** ***Fermez fort les yeux***			**Zygomatiques** ***Souriez fort***		
	Nb de mouvements par série jusqu'à la fatigue			Nb de mouvements par série jusqu'à la fatigue			Nb de mouvements par série jusqu'à la fatigue		
	Série 1	Série 2	Série 3	Série 1	Série 2	Série 3	Série 1	Série 2	Série 3
J151									
J152									
J153									
J154									
J155									
J156									
J157									
J158									
J159									
J160									
J161									
J162									
J163									
J164									
J165									
J166									
J167									
J168									
J169									
J170									
J171									
J172									
J173									
J174									
J175									
J176									
J177									
J178									
J179									
J180									
