# Supplementary material for: Guided Self-rehabilitation Contract vs conventional therapy in chronic peripheral facial paresis: VISAGE, a multicenter randomized controlled trial
Source: BMC Neurol. 2023 Apr 10;23:148. doi: 10.1186/s12883-023-03096-8 (PMC10084642; doi:10.1186/s12883-023-03096-8)
Supplement: Supplementary file 3 — Additional file 3: Appendix C. Rehabilitation booklet of the conventional therapy group. [file 12883_2023_3096_MOESM3_ESM.pdf]

Carnet de suivi

VISAGE

Groupe Kinésithérapie ou orthophonie

Effet clinique, cinématique et sur la qualité de vie de deux programmes de rééducation dans la parésie faciale périphérique chronique

Les coordonnées de votre médecin figurent à la fin de ce carnet.

Votre identifiant dans l'étude : **I__I__I__I - I__I__I__I__I - I__I__I**
(n° de centre, n° d'inclusion, Initiale du nom, Initiale du prénom)

Nom de l'investigateur:

Adresse:

Préambule

Madame, Monsieur,

L'objectif de l'étude à laquelle vous avez accepté de participer est de comparer deux programmes de rééducation dans la récupération motrice d'une parésie faciale périphérique unilatérale au stade chronique.

Lors de la visite d'inclusion, vous avez été tiré au sort dans le groupe rééducation par kinésithérapie et/ou orthophonie. Ce groupe implique la prescription d'une ordonnance de kinésithérapie et/ou orthophonie et le suivi en externe de votre parésie faciale de façon régulière durant les 6 mois de rééducation de l'étude. La fréquence des séances ainsi que leur contenu sont laissées au choix de chaque thérapeute de ville, en concertation avec vous-même.

Il est important d'apporter ce carnet à chaque visite.

Consignes importantes

→ Il est nécessaire que les hommes effectuent un rasage la veille au soir ou le matin des 3 demi-journées d'évaluation pour ne pas gêner les évaluations.

→Il est nécessaire que les femmes ne se maquillent pas ou ne portent pas de crème le matin des 3 demi-journées d'évaluation pour ne pas gêner les évaluations.

→Le jour des 3 visites (J0, J90 et J180), il est nécessaire de porter un haut de couleur noire et de retirer tous les bijoux au niveau du visage, du cou et des oreilles.

→ Il est important de poursuivre votre rééducation tout au long de l'étude.

Rappel des traitements interdits durant l'étude

Durant l'étude, certains traitements sont susceptibles de compromettre les évaluations.

Il est important de ne pas avoir recours aux traitements suivants durant votre participation à l'étude :

- Injections de toxine botulique au visage
- Injections d'acide hyaluronique au visage
- Injections de graisse au visage
- Laser au visage
- Chirurgie au visage

Visite d’inclusion : J-30

- Elle a lieu 30 jours avant le début des évaluations
- Durée : 30 minutes
- Dans un des 5 centres d’inclusion de l’étude
- Déroulement :
  - Explication de l‘étude VISAGE
  - Réponses aux questions éventuelles
  - Vérification des critères d’inclusion et de non-inclusion
  - Remise de la note d’information et signature du consentement
  - Inclusion et randomisation dans l’étude
  - Remise de votre carnet-patient à ramener à chaque journée d’évaluation : J0, J90 et J180
- La date de la première évaluation, c’est-à-dire J0, vous sera communiquée dans les jours suivants par un appel téléphonique donné par le service de Rééducation Neurolocomotrice de l’hôpital Henri Mondor de Créteil (centre évaluateur de l’étude).

Appels téléphoniques

- Durée : 5 minutes par appel
- Vous recevrez 4 appels téléphoniques brefs réalisés par le Dr Gault-Colas, médecin rééducateur de l’étude
- Les appels seront programmés selon le calendrier suivant :
  - J30
  - J60
  - J120
  - J150

- Ils permettront de vérifier la poursuite de votre rééducation
- Le jour et l’heure de chaque appel sera décidé conjointement entre le Dr Gault-Colas et vous.

Evaluations : J0 – J90 – J180

Les 3 évaluations auront exclusivement lieu à l'adresse suivante :

Service de Rééducation Neurolocomotrice de l'hôpital Henri Mondor
Rez de chaussée bas – 51 av du M^{al} de Lattre de Tassigny – 94010 Créteil

1ère évaluation - J0 :

- Durée : 2h30
- **Apporter le carnet-patient**
- Consultation d'évaluation : échelles cliniques, évaluation 3D, auto-questionnaires
- Consultation de rééducation : prescription de la rééducation

2ème évaluation - J90 :

- Durée : 1h30
- **Apporter votre carnet-patient**
- Consultation d'évaluation : échelles cliniques, auto-questionnaires ;
- Consultation de rééducation : vérification de la bonne réalisation de la rééducation.

3ème évaluation - J180 :

- Durée : 2h30
- **Apporter votre carnet-patient**
- Consultation d'évaluation : échelles cliniques, évaluation 3D, auto-questionnaires ;
- Consultation de rééducation : vérification de la bonne réalisation de la rééducation.

Agenda de votre participation à l'étude

	**J-30** **Inclusion**	**J0** **Evaluation 1**	J30 Appel 1	J60 Appel 2	**J90** **Evaluation 2**	J120 Appel 3	J150 Appel 4	**J180** **Evaluation 3**
Date								
Note d'information	X							
Signature du consentement	X							
Vérification critères de sélection	X							
Randomisation	X							
Prescription de la rééducation		X						
Echelles cliniques	X	X			X			X
Auto-questionnaires		X			X			X
Analyse 3D		X						X
Recueil effets indésirables					X			X
Appels téléphoniques			X	X		X	X	

Contacts

En cas d'impossibilité de vous rendre à une des évaluations, merci de prévenir le service aux coordonnées téléphoniques suivantes :

- Secrétariat médical – Mme COOLS – Hôpital Mondor : 01 49 81 24 98 ;
- Accueil du service – Hôpital Mondor : 01 49 81 24 97 ;
- Secrétariat médical – Mme BLONDEL – Hôpital Chenevier : 01 49 81 30 61.

En cas de nécessité ou de problème médical, vous pouvez joindre le Docteur Dr GAULT-COLAS, médecin de rééducation de l'étude au courant de votre groupe de traitement, aux coordonnées suivantes :

Service de Rééducation Neurolocomotrice - Hôpital Henri Mondor de Créteil

Secrétariat téléphonique : 01 49 81 24 98 - 01 49 81 24 97 - 01 49 81 30 61

En cas d'absolue nécessité et si les numéros ci-dessus ne fonctionnent pas, vous pouvez joindre le Dr BAUDE, médecin coordinateur principal de l'étude aux coordonnées suivantes :

Service de Rééducation Neurolocomotrice - Hôpital Henri Mondor de Créteil

Secrétariat téléphonique : 01 49 81 24 98 - 01 49 81 24 97 - 01 49 81 30 61

Adresse mail : marjolaine.baude@aphp.fr
